# Rifampicin-induced thrombocytopenia

**DOI:** 10.4103/0253-7613.68432

**Published:** 2010-08

**Authors:** Ajay Kumar Verma, Arpita Singh, Amol Chandra, Santosh Kumar, Rajesh Kumar Gupta

**Affiliations:** Department of TB and Chest Diseases, S.N. Medical College, Agra - 282 002, India; 1 Department of Pharmacology, GSVM Medical College, Kanpur - 208 002, India

**Keywords:** Rifampicin, thrombocytopenia, tuberculosis

## Abstract

In the treatment of tuberculosis there are special therapeutic problems related to adverse effects of drugs, compliance to treatment, and microbial resistance. Thrombocytopenia is an uncommon but potentially fatal adverse effect of certain anti-tubercular drugs when the incriminating drug is taken by a susceptible individual. We report a case of rifampicin-induced thrombocytopenia, which although rare, needs attention.

## Introduction

Treatment of tuberculosis has been a therapeutic challenge since long. Most anti-tubercular drugs are relatively safe but serious reactions are not uncommon. Adverse reactions due to rifampicin are either dose related or allergic. Thrombocytopenia is an uncommon but potentially fatal adverse effect seen with certain anti-tubercular drugs, including rifampicin.[1] To identify the offending agent in a patient taking several medications, it poses a challenging clinical problem.[2,3] Confirmation of drug-induced thrombocytopenia at the time of initial presentation is not often possible as tests for drug-dependent anti-platelet antibodies are not available in most laboratories.[4] Discontinuation of suspected drug leading to resolution of thrombocytopenia provides a strong evidence of drug-induced thrombocytopenia. Rifampicin-induced thrombocytopenia was first reported in 1970.[5,6] It is usually reversible if detected early and treated appropriately. Other drugs known to cause thrombocytopenia are quinine, quinidine, chloroquine, sulfonamides, tolbutamide, chlorothiazide, digoxin, penicillamine, amphotericin B, sedatives, anticonvulsants, methyldopa, aspirin, etc.[6]

## Case Report

**Figure 1:**
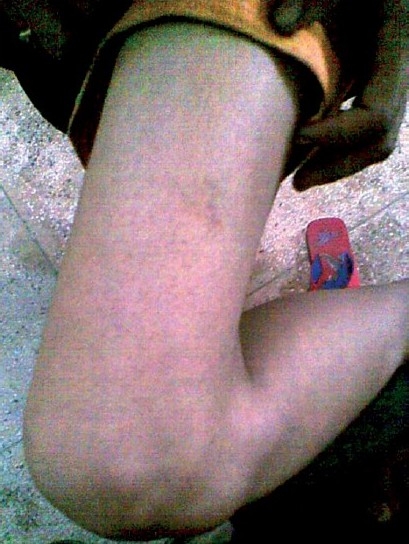
Purpura on the ateriolateral aspect of thigh

**Figure 2:**
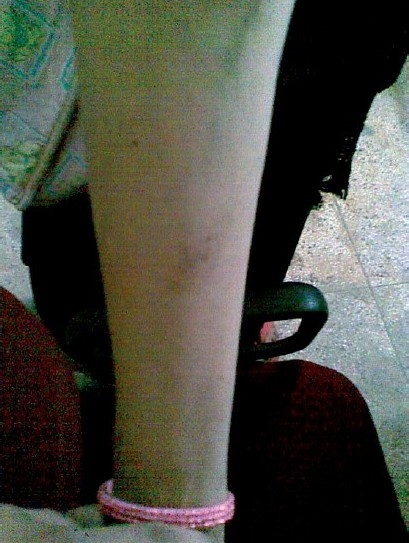
Purpura on the flexor surface of arm.

A 40-year-old female, health care worker, presented to the Department of Tuberculosis and Chest Diseases, S.N. Medical College Agra (U.P.), with chief complaints of low grade fever and a swelling in the sub-mental region since 1 year. The swelling was very hard in consistency. The patient was prescribed anti-tubercular treatment (ATT) by a private practitioner in Agra that included rifampicin (R), isoniazid (H), pyrazinamide (Z), and ethambutol (E) for 1 month, daily. She was later started on ATT DOTS III and completed the full course of recommended treatment category. The patient, however, did not respond to the treatment. After this she consulted a private practitioner who prescribed R, H, Z, E, and streptomycin (Sm). Following 10 days of this, she presented to the outpatient department (OPD) with complaint of purpura over her right anterolateral part of thigh [Figure 1] and over the flexor surface of her right forearm [Figure 2].

At the time of this presentation, a clinical suspicion of rifampicin-induced thrombocytopenia was made. The patient was investigated accordingly. Blood examination revealed hemoglobin of 12 g%, serum uric acid of 6.9 mg%, anti-nuclear antibodies titer was 0.72 (i.e., negative), total leucocyte count (TLC) of 5,800 per mm^3^, differential leucocyte count (DLC, N---43%, L---45%, E---09%,B---02%) and platelet count of 0.60 million per mm^3^. Chest skiagram revealed no abnormality. Her FNAC from the submental swelling was positive for acid fast bacilli (AFB) by Ziehl Neelsen staining. Rifampicin was withdrawn. Following about 2 weeks from this, the patient was again investigated and her investigations revealed hemoglobin of 12 g%, TLC of 8,800 per mm^3^, DLC N---73%, L---25%, E---00, M---02%, B---00. Blood picture showed normocytic, normocromic RBC with adequate platelets, no hemoparasites (platelet count of 2.4 million per mm^3^). The patient also complained of pain in her knee joints along with tingling and numbness in the distal extremities and ringing of the ears. Following these complaints streptomycin was also withdrawn and she was prescribed a regimen consisting of H, Z, E, and levofloxacin, along with analgesics. There were no similar complaints except the submental swelling on subsequent follow up visits. The swelling was reduced in size, signifying a response to drug therapy. The patient continued the same therapy and responded well.

## Discussion

Thrombocytopenia can occur with any of the primary anti-tubercular drugs. In case of isoniazid, it occurs as a hematological reaction.[7] Ethambutol[8] and pyrazinamide[9] have also been reported to cause thrombocytopenia, perhaps by an immunological mechanism.

Adverse reactions to rifampicin are uncommon on daily regimens but are relatively common with intermittent regimens.[4] These include cutaneous syndrome, abdominal syndrome, a flu like syndrome, respiratory syndrome, purpura, and elevated transaminase serum levels.[10] Thrombocytopenia is an adverse reaction associated with intermittent rifampicin regimen. Our patient had thrombocytopenia on daily rifampicin which was re-started 4 months after she had last received rifampicin. Rifampicin was omitted after a clinical suspicion of drug-induced thrombocytopenia was made.

Pool *et al*.[11] reported a highly significant correlation between the presence of rifampicin-dependent anti-bodies and the occurrence of adverse reactions (with 1200 mg of rifampicin, twice weekly). They recorded three cases of thrombocytopenia. The risk of purpura occurring with daily rifampicin cannot be completely discounted. The drugs causing thrombocytopenia lead to either suppression of platelet production or immunologic platelet destruction. Most drugs induce thrombocytopenia by the latter mechanism. The platelets are damaged by complement activation following the formation of drug--antibody complex. Current laboratory tests can identify the causative agent in 10% of patients with clinical evidence of drug-induced thrombocytopenia. However, the best proof of a drug-induced etiology is a prompt rise in the platelet count when the suspected drug is discontinued.[5]

According to *WHO-UMC Causality Categories*, the association of rifampicin as the causal drug for this ADR is “Probable / likely”.[12]

Ferguson and the Hong Kong trial reported TCP even with daily rifampicin.[6] Incidence of TCP occurred from the 1^st^ to the 14^th^ month of therapy on rifampicin.[1] Most workers agree that continuous treatment with rifampicin results in neutralization of any of the antibodies formed, the antigen--antibody complex being continuously removed without causing allergic reaction. Discontinuation of treatment allows a sufficient quantity of antibody to be built up during the drug-free interval so that when rifampicin is re-administered, an intense reaction ensues. *In vitro* tests, for identifying circulating antibodies are not easy to perform. No single test (complement fixation test, immuno injury test) detects all the cases of drug-induced TCP. Direct binding assays for IgG or complement on the platelet surface are very useful.[4] Although most patients recover within 7 to 10 days and do not require therapy, occasional patients with platelet counts below 10,000 to 20,000 per *μ*l have severe hemorrhage and may require temporary support with glucocorticoids, plasmapheresis, or platelet transfusions. Reuse of the offending drug has to be avoided in the future since only minute amounts of drug are needed to set up subsequent immune reactions.[6] If purpura occurs, rifampicin should be stopped immediately and should not be given again even in small doses.[8]
